# Underlying mechanisms of visual mismatch responses – An EEG-fMRI study

**DOI:** 10.1016/j.isci.2025.114039

**Published:** 2025-11-17

**Authors:** Insa Schlossmacher, Ina Protmann, Jacky Dilly, David Hofmann, Torge Dellert, Antje Peters, Marie-Luise Roth-Paysen, Robert Moeck, Maximilian Bruchmann, Thomas Straube

**Affiliations:** 1 Institute of Medical Psychology and Systems Neuroscience, University of Münster, 48149 Münster, Germany; 2 Otto Creutzfeldt Center for Cognitive and Behavioral Neuroscience, University of Münster, 48149 Münster, Germany

**Keywords:** Neuroscience, Sensory neuroscience, Cognitive neuroscience

## Abstract

Unexpected compared to expected visual events are accompanied by stronger neural responses. Two key mechanisms for these heightened responses are prediction errors to oddballs and adaptation to frequent stimuli. Whether these mechanisms vary systematically between early and late responses or differentlt in different cortical areas is yet unknown. In the current study, we simultaneously measured EEG and fMRI during an oddball task with control conditions tailored to distinguish between the different mechanisms. Results showed visual mismatch negativity and an enhanced P3 in the EEG and increased activations in the occipital cortex (OC) and superior parietal lobe (SPL) in fMRI. While adaptation predominantly drove early mismatch responses in the EEG and posterior OC, responses at later time points and in the anterior OC and SPL could best be explained by prediction error. This pattern suggests that the relative contribution of different mechanisms to deviance processing varies systematically in time and space.

## Highlights

• We measured BOLD activity and ERPs during a visual oddball and control sequence
• Early ERPs and sensory regions are characterized by adaptation
• Late ERPs and sensory and higher-order regions are linked to a prediction error
• Results are in line with a hierarchical organization of the predictive brain

## Graphical abstract

**Figure:**
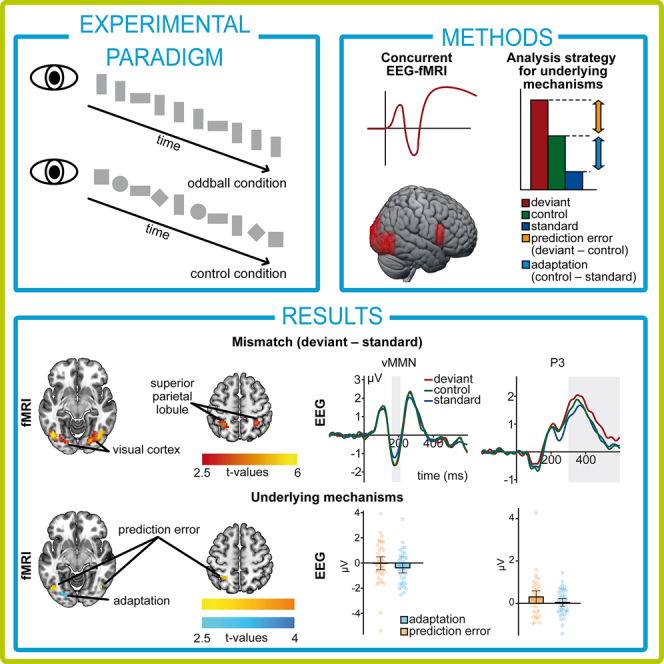

## Introduction

Detecting visual changes in our environment is a cornerstone of perception. An increasing body of evidence has shown that neural responses to visual deviance in oddball studies are associated with increased activation in electroencephalography (EEG) and functional magnetic resonance imaging (fMRI) studies for reviews, see.^1^^,^^2^^,^^3^^,^^4^^,^^5^ The underlying mechanisms of these increased responses are only partially understood. Hierarchical predictive processing has been put forward as a theoretical framework for these effects.^4^^,^^6^^,^^7^^,^^8^ From this point of view, the increase in neural activation for unexpected rare stimuli compared to expected frequent ones stems from a process where a prediction of the next input is compared with the actual sensory input. If prediction and input do not match, as is the case when a deviant stimulus is presented, a prediction error (PE) signal would be elicited. This PE would then be propagated upwards in the hierarchy and compared with the predictions at the next higher level and so forth, enabling efficient information processing.^4^^,^^6^

However, while the predictive processing offers a compelling explanation for deviance-related effects, it is not the only process that could be responsible for the observed difference between rare and frequent stimuli. Adaptation-related processes have also been proposed to play an important role during deviance processing.^9^^,^^10^^,^^11^ In this theoretical framework, at least early neuronal differences between rare and frequent stimuli stem from reduced neuronal responsiveness to the frequent stimulus, which thus elicits a smaller response compared to the non-adapted cells (fresh afferents) activated by the rare stimulus. In other words, some deviance responses in typical oddball designs are not driven by genuine mismatch responses but by altered, i.e., reduced, responses to the standard stimulus.

At first glance, the PE and the adaptation-related account seem opposing. However, there is evidence that both processes can be present in the brain to variable degrees in the same region or at the same time.^12^^,^^13^^,^^14^^,^^15^ One way to delineate the contribution of adaptation and PE to deviance effects is to compare the responses of both deviant and standard stimuli to a suitable control stimulus.^15^^,^^16^^,^^17^ Using this approach, there is initial evidence from fMRI, intracranial EEG, and animal models in audition that the relative contribution of PE vs. adaptation increases along the auditory processing hierarchy, i.e., areas lower in the hierarchy show more prominent adaptation while the PE increases in higher-order areas.^13^^,^^14^^,^^15^^,^^18^^,^^19^ In the EEG, the mismatch negativity (MMN), a negative component between 100 and 300 ms, is affected by both adaptation and PE,^16^^,^^20^^,^^21^^,^^22^^,^^23^^,^^24^ while later occurring EEG deviance signals, such as the P3, have been associated with a prediction error.^25^^,^^26^ Thus, both signals might be associated with different hierarchical processing levels in the brain.

In the visual modality, no fMRI studies that could delineate adaptation and PE have been conducted yet. However, generally increased responses to deviants have been observed in areas involved in sensory processing, such as the visual cortex, as well as higher-order areas, including the inferior frontal junction, anterior insula, and superior parietal cortex.^1^^,^^27^ Visual EEG studies with control designs or computational modeling show that both adaptation and prediction error drive the visual MMN vMMN,^28^^,^^29^^,^^30^ even though adaptation seems the prominent mechanism if oddballs are unattended and task-irrelevant.^31^ While there are no studies that could separate the role of adaptation or PE for the P3, several lines of evidence suggest that the P3 responses are strongly driven by PE computations.^25^^,^^26^^,^^32^^,^^33^^,^^34^

Thus, it remains to be investigated using fMRI in the visual modality, where adaptation and PE-related processes can be observed in the brain. Furthermore, the question arises of how these brain activations relate to adaptation- or PE-driven modulations of typical deviance-dependent ERP components such as the MMN or the P3. In order to investigate the latter question, combined EEG-fMRI is especially suited. This approach allows the direct comparison of the contributions of each mechanism in the spatial and temporal domains in the same dataset. To our knowledge, such a study has not been reported yet, neither in the visual nor in the auditory or any other sensory modality.

Therefore, the present study investigated adaptation and PE mechanisms in the visual modality using concurrent EEG-fMRI and a suited control condition with equiprobable stimuli. This allowed contrasting the deviant to a physically identical control and the control to the standard to delineate PE-related and adaptation-related activity, respectively. Following prior research in the auditory domain,^15^^,^^18^^,^^19^ we hypothesized from the fMRI data that on lower levels of the cortical hierarchy, such as early occipital areas, adaptation-related activity would be more prominent, while on higher levels (e.g., higher levels of occipital cortex, superior parietal cortex, inferior frontal junction, anterior insula), activity should be driven by PEs. Correspondingly, for the EEG, we predicted that the vMMN would best be explained by adaptation and the P3 by prediction error. In addition to the separate analysis of EEG and fMRI data, we tried to relate both neuroscientific methods to each other by correlating individual differences in ERP and fMRI contrasts regarding deviance and possible adaptation and PE effects. We expected that the vMMN and ERP adaptation effects would covary with activation in early sensory regions, while the P3 and PE-related ERP effects would covary with both sensory regions and higher-order areas.

## Results

### Participants complied with the task

**Figure 1:**
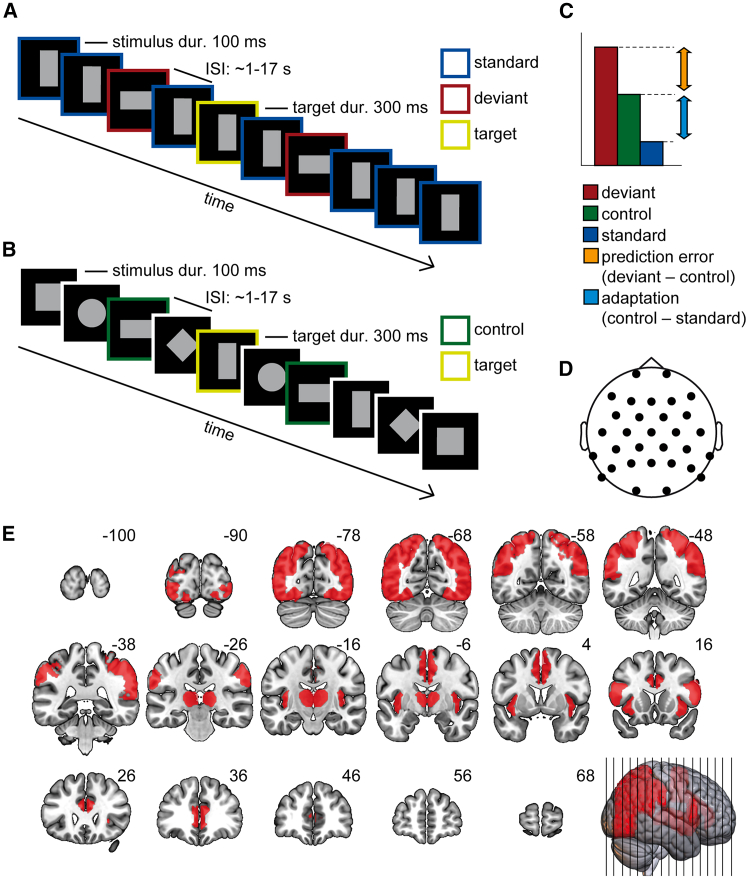
Experimental paradigm and mask for data analysis (A) Schematic of oddball sequence. (B) Schematic of control sequence. Which rectangle served as the deviant was counterbalanced across participants in the experiment. (C) Decomposition of observed responses into prediction error-related and adaptation-related activity. (D) Electrode placement. (E) Illustration of the mask applied during the cluster-based permutation test. Red areas were included in the mask. The image was generated using MRICroGL.

Participants viewed oddball and control sequences made of geometrical shapes while performing a duration estimation task unrelated to the oddball sequence (Figures 1A and 1B). During the task, participants had to respond when a long-duration target appeared. Performance on the duration task was high, indexed by an average hit rate of 0.89 (SD = 0.12), an average false-alarm rate of 0.01 (SD = 0.02), and an average *d’* of 3.77 (SD = 0.73), indicating that participants were able to comply with the task easily.

### Systematic variation in prediction error and adaptation in the spatial domain

**Figure 2:**
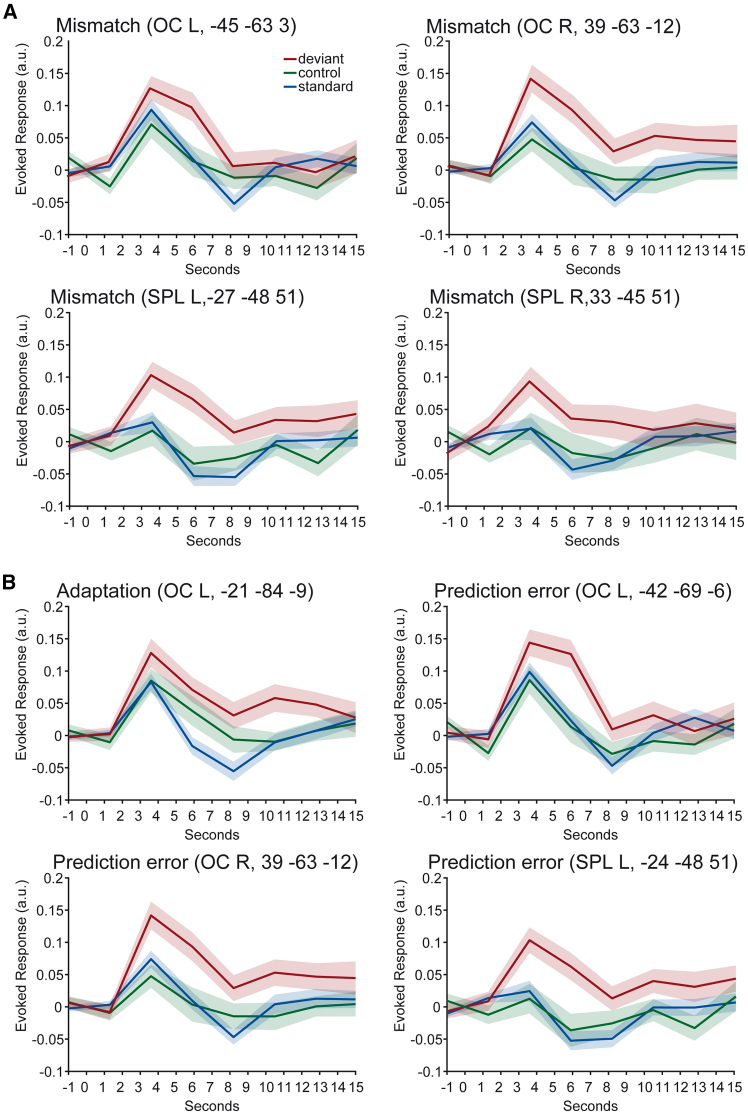
Visualization of hemodynamic responses (A) BOLD time courses for mismatch contrasts at peak voxels in the left and right OC and SPL. (B) BOLD time courses at peak voxels for adaptation and prediction error clusters. The upper left plot corresponds to the adaptation peak voxel, and all three remaining plots to prediction error peak voxels. Shaded area represents standard error of the mean.

**Figure 3:**
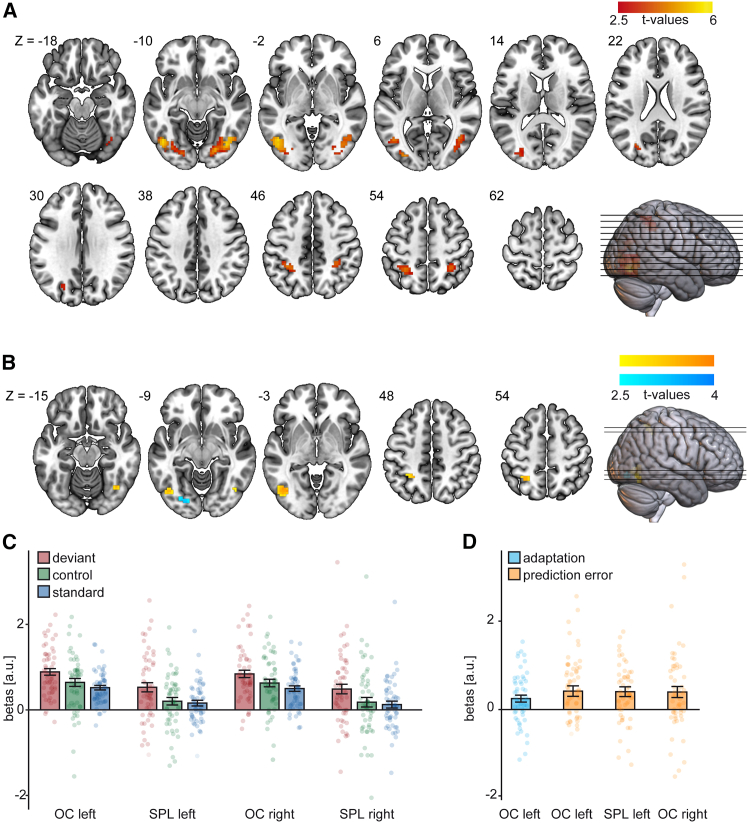
Visual mismatch responses and underlying mechanisms measured by fMRI (A) Clusters found in the cluster-based permutation (CBP) comparing deviant and standard stimulus. (B) Clusters found in the CBP investigating adaptation (control – standard) and prediction error (deviant – control). (C) Mean beta values extracted from the significant mismatch clusters. The deviant, standard, and control stimuli are displayed. (D) Mean beta values extracted from the significant adaptation and prediction error clusters. Average values of the difference betas are displayed. OC: occipital cortex, SPL: superior parietal lobe. Dots represent single participants. Error bars represent standard errors of the mean. Images in (A) and (B) were generated using MRICroGL.

In fMRI, the cluster-based permutation test revealed bilateral clusters of significant mismatch processing (deviant – standard) in the occipital cortex (left: *p* = 0.002; right: *p* = 0.002) including mainly voxels in inferior lateral occipital cortex (left: 58.09%; right: 49.32%), fusiform occipital cortex (left: 19.14%; right: 40.82%) and superior lateral occipital cortex (left: 15.84%; right: 0.34%). Furthermore, two clusters were found in the parietal cortex (left: *p* = 0.020; right: *p* = 0.035), including mainly voxels in the SPL (left: 92.78%; right: 91.89%). Please see Figure 2 for a visualization of BOLD time courses, Figure 3 for a visualization of clusters and beta-values, and Table 1 for the peak coordinates, *t*-statistics, and number of voxels (k) of the effects.

**Table 1:** fMRI results of the oddball, adaptation, and prediction error contrast

Contrast	Area	Hemisphere	x	y	z	max(*t*)	mean(*t*)	k
Mismatch	OC	L	−45	−63	3	5.67	3.81	303
	OC	R	39	−63	−12	5.48	3.66	294
	SPL	L	−27	−48	51	4.47	3.37	97
	SPL	R	33	−45	51	4.04	3.22	74
Adaptation	OC	L	−21	−84	−9	3.23	2.89	18
Prediction error	OC	L	−42	−69	−6	3.85	3.16	69
	OC	R	39	−63	−15	3.52	3.00	21
	SPL	L	−24	−48	51	3.60	3.10	31

OC: occipital cortex, SPL: superior parietal lobule. x, y, and z correspond to peak MNI coordinates

As the cluster-based permutation test does not allow quantifying the evidence for the absence of an effect, we computed Bayes factors for betas from 8 mm spheres at MNI peaks reported in the meta-analysis of Kim.^1^ Results indicated evidence for the absence of a mismatch effect in the AI, IPL, TPJ, and thalamus in the left and right hemispheres, respectively (BF_01_ > 3) and inconclusive evidence in the left and right IFJ and ACC/SMA (1.88 < BF_01_ < 2.44).

For the adaptation contrast (control – standard), the cluster-based permutation test yielded one significant cluster in the OC (*p* = 0.036). Concerning the prediction error contrast (deviant – control), three significant clusters were found in the left (*p* = 0.003) and right (*p* = 0.027) OC and left SPL (*p* = 0.016). See Table 1 and Figure 3. Two prediction error clusters in the right SPL (*p* = 0.131, *p* = 0.111) and one in the left SPL (*p* = 0.115) failed to reach significance.

### Different underlying mechanisms for early and late event-related potential effects

**Figure 4:**
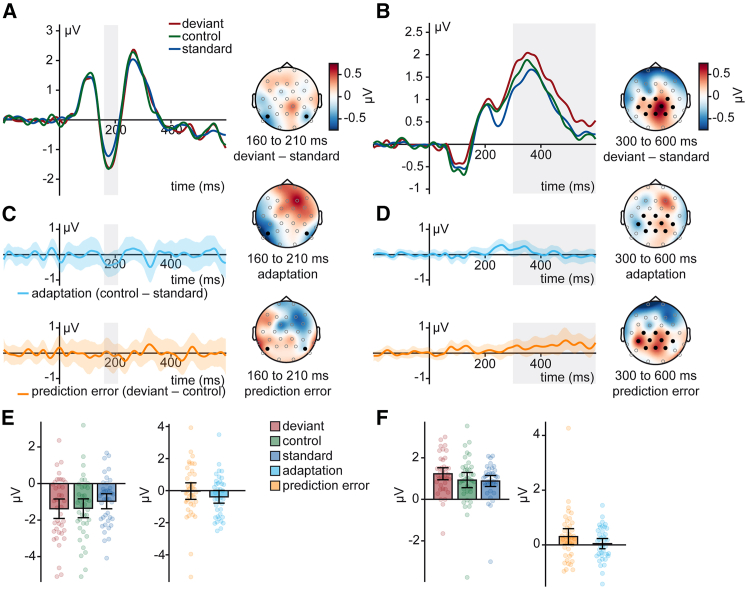
Visual mismatch responses and underlying mechanisms measured by EEG Wavefoms and mismatch topography between 160 and 210 ms (A) and from 300 to 600 ms (B) from electrodes marked in bold. The time window of interest is marked in gray. Difference waveforms and topographies representing adaptation and prediction error in the vMMN (C) and P3 (D) time window. Bar plots of averaged amplitudes in the vMMN (E) and P3 (F) time window. Dots represent single participants. Error bars represent standard errors of the mean. The shaded area around ERP waveforms represents the 95%-bootstrap confidence interval.

Exclusion based on the SNR results in 39 participants with sufficient data quality. From these 39 participants, on average, 0.62 (SD = 1.33) electrodes were interpolated, and 22.74% (SD = 11.93) of trials were excluded. We found a significant vMMN effect between 160 and 210 ms at electrodes P7 and P8 (*t*(38) = −2.20, *p* = 0.017, BF_10_ = 2.89), see Figure 4A. Furthermore, a significant P3 effect was found between 300 and 600 ms at central electrodes (*t*(38) = 2.82, *p* = 0.004, BF_10_ = 10.34), see Figure 4B.

We again used an average amplitude approach to investigate adaptation-related and prediction-error-related activity, see Figures 4C and 4D. We found significant evidence for adaptation-related processes in the vMMN time window (*t*(38) = −1.86, *p* = 0.036, BF_10_ = 1.57) while we observed evidence for the absence of a prediction error-related effect in this time window (*t*(38) = −0.10, *p* = 0.462, BF_01_ = 5.37). For the P3, however, we observed significant evidence for prediction error-related processes (*t*(38) = 2.02, *p* = 0.025, BF_10_ = 2.09), while we observed evidence for the absence of an adaptation-related effect in this time window (*t*(38) = 0.48, *p* = 0.317, BF_01_ = 3.84).

### Correlation of electroencephalography and functional magnetic resonance imaging data across subjects reveals associations for mismatch contrasts but not prediction error and adaptation contrasts

**Figure 5:**
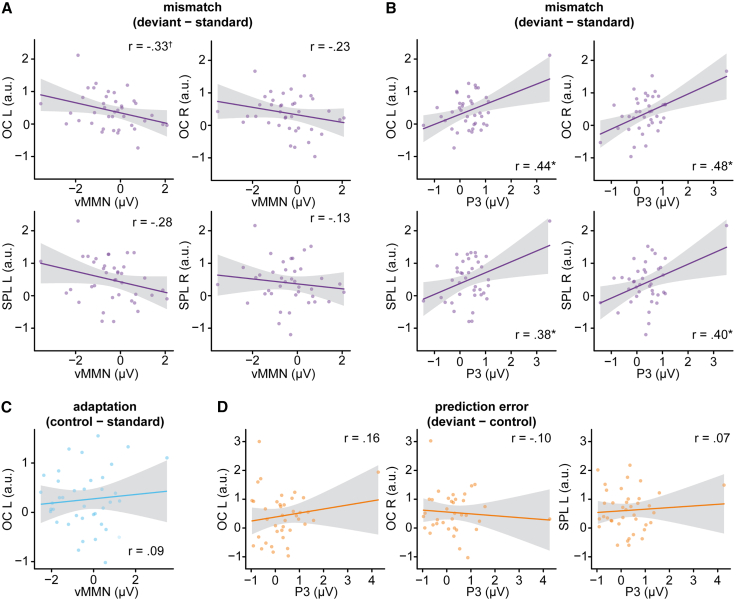
Correlations between fMRI and EEG activity (A) Correlations between the averaged amplitude difference (deviant – standard) in the vMMN time window and mismatch clusters. (B) Correlations between the averaged amplitude difference (deviant – standard) in P3 time window and mismatch clusters. (C) Correlation between averaged amplitude difference (control – standard) in vMMN time window and adaptation cluster. (D) Correlations between averaged amplitude difference (deviant – control) in the P3 time window and prediction error clusters. Single dots represent the values of single participants. †*p* < 0.1, ∗*p* < 0.05, ∗∗*p* < 0.01 by Pearson’s product-moment correlation corrected for multiple comparisons using FDR correction. Shaded areas represent confidence bands.

Correlating vMMN mismatch difference amplitudes with mismatch beta-differences from OC and SPL clusters yielded, after correction for multiple comparisons, a marginally significant correlation for the left OC (*r* = −0.33, *t*(37) = −2.13, *p*_*FDR*_ = 0.095, BF_10_ = 2.29). Please note that negative correlations between negative ERPs and positive BOLD responses indicate a positive relationship. Correlations with betas from the three remaining regions did not reach significance, but Bayesian evidence against an effect remained inconclusive (all *r* < −0.27, all *p*_*FDR*_ > 0.17, 0.76 < BF_01_ < 2.01), see Figure 5A. Correlating P3 mismatch difference amplitudes with mismatch beta-differences from OC and SPL clusters yielded significant correlations for all four regions (left OC: *r* = 0.44, *t*(37) = 2.96, *p*_*FDR*_ = 0.031, BF_10_ = 10.64; right OC: *r* = 0.48, *t*(37) = 3.36, *p*_*FDR*_ = 0.022, BF_10_ = 25.26; left SPL: *r* = 0.38, *t*(37) = 2.53, *p*_*FDR*_ = 0.046, BF_10_ = 4.62; right SPL: *r* = 0.40, *t*(37) = 2.69, *p*_*FDR*_ = 0.043, BF_10_ = 6.18), see Figure 5B. Concerning relationships between different mechanisms in EEG and fMRI, we correlated adaptation and prediction error amplitudes of vMMN and P3 with the betas recovered from prediction error and adaptation clusters. No correlations reached significance, but evidence for the absence of effects remained inconclusive (all *p*_*FDR*_ > 0.517, 1.19 < BF_01_ < 2.75), see Figures 5C and 5D.

## Discussion

In this study, we investigated the contributions of adaptation and prediction errors to explain increased deviance-related brain activation in humans. The fMRI data showed enhanced mismatch responses in the occipital cortex and superior parietal cortex. In the posterior occipital region, these responses could mainly be explained by adaptation-related processes, while the more anterior occipital and superior parietal regions were rather driven by prediction error processing. In the EEG, deviants vs. standards led to a vMMN and enhanced P3 component. While the vMMN was primarily driven by adaptation-related processes, a prediction error account was predominantly observed for the P3.

Comparing oddball and standard stimuli in the fMRI data confirmed deviance-related activation in ROIs that are part of the visual processing hierarchy. This includes the occipital cortex and the superior parietal lobule. Based on the parcellation of Yeo et al.,^35^ the majority of voxels in the occipital clusters belong to the visual network (left: 68%, right: 82%). This is in line with shape information being processed in the lateral occipital and occipital fusiform areas.^36^^,^^37^^,^^38^^,^^39^^,^^40^ On a descriptive level, we observed an involvement of the superior lateral occipital cortex only in the left hemisphere, which is seemingly at odds with studies of shape processing indicating no hemispheric differences.^41^^,^^42^ However, as we did not expect hemispheric differences, the difference in the involvement of superior occipital areas between the hemispheres was not tested statistically. Furthermore, lowering the voxel-wise thresholds (*p* = 0.05) would also reveal effects in the right hemisphere, suggesting that the absence of right-sided effects represents a threshold effect.

The activation of the SPL has been observed in various experimental paradigms during expectancy violations.^43^^,^^44^ The deviance-related effects in bilateral SPL are in line with its role in the dorsal attention network, possibly indicating the orienting of attention to the deviant, even though no explicit task was required.^35^^,^^45^^,^^46^ Besides the role of the posterior parietal cortex in visuospatial attention see also,^47^^,^^48^ there is also evidence of its involvement in the generation of object representations^49^ and decision making.^50^^,^^51^^,^^52^

Taken together, we observed mismatch responses in the OC, which is more tightly associated with sensory processing, as well as in the SPL, an area commonly linked to higher-order processes see also.^50^ In other areas, evidence for the absence of deviance-related effects or inconclusive evidence was observed, including the inferior frontal junction or anterior insula, which are areas commonly activated in deviance processing.^1^^,^^27^ One possible explanation for the lack of effects could be that the deviance elicited by the vertical and horizontal rectangles was not salient enough to evoke activity in these regions, which have been linked to the ventral attention network,^1^^,^^46^ or that our analysis was too conservative to detect potential effects.

Deviance-related effects in LOC and SPL could be explained to varying degrees by adaptation- and prediction error-related activity. We found significant contributions of adaptation in the left posterior LOC and more anterior clusters of prediction-error-related activity in the left and right LOC. This finding could be linked to a gradient of processing mechanisms similar to the auditory modality.^13^^,^^14^^,^^19^ These gradients fit well with the idea of a hierarchical posterior-anterior flow of information from perceptual to more general conceptual representations.^53^ Furthermore, in the left SPL, a significant prediction error effect was found, but no significant adaptation effect, which is in line with parietal areas being implicated in several meta-analyses on prediction violation.^43^^,^^54^ While no fMRI studies with suited control stimuli to delineate adaptation and PE in the visual modality existed so far, a computational modeling study reported effects of PE in several regions, including visual areas, the thalamus, the cerebellum, and the inferior parietal lobule during a task-irrelevant roving oddball paradigm.^55^ However, no comparison to an adaptation model has been reported. Thus, no conclusions on the relative contribution of both models can be drawn.

In the EEG, we observed significant early and late correlates of deviance processing, namely the vMMN and an enhanced P3. The vMMN represents automatic change detection and can at least in part be elicited even if stimuli are unattended and not consciously perceived.^4^^,^^31^^,^^56^ It has been suggested that the vMMN primarily represents activation in visual cortex areas.^32^^,^^57^^,^^58^ The P3 in oddball designs has been shown to be influenced by various factors such as local and global stimulus probability, novelty, motivational significance, salience, and attention devoted to the stimuli.^3^^,^^56^^,^^59^ The P3 is believed to reflect the activation of different sources, including parietal and temporo-occipital activation.^50^^,^^60^^,^^61^^,^^62^

In the EEG data, the prominent mechanisms, as uncovered in the control paradigm, were adaptation in the early time window and prediction error in the late time window. The presence of an early adaptation effect is in line with several studies reporting it as a main contributor to the vMMN.^63^^,^^64^^,^^65^ However, several studies also found a contribution of prediction error responses to the vMMN.^28^^,^^29^^,^^30^^,^^66^ PE-related processes in the MMN seem to increase with task-relevance of stimuli, while the MMN is more strongly driven by adaptation processes in unaware or task-irrelevant conditions.^31^ In the current study, at least the oddball structure was completely task-irrelevant. Furthermore, we cannot exclude that we did not see prediction-error-related responses during the early time window due to the noisy scanner environment and specific electrode configurations for the scanner. In contrast to the MMN, the P3 was better explained by PE, which is in line with accounts that propose the P3 as a prediction error response at a later stage in the processing hierarchy^25^^,^^26^ and agrees with computational modeling studies.^32^^,^^33^^,^^34^

Thus, both EEG and fMRI data indicate that mismatch at earlier time points and in posterior occipital areas are driven by adaptation, and later time points and higher-order areas are driven by prediction error. This general pattern of results is in line with a hierarchical account in the predictive processing framework, with an increasing prevalence of prediction-related processes in higher-order areas or at later time points.^7^^,^^15^^,^^19^ Combining the EEG and fMRI data, however, revealed only some significant associations between ERP and fMRI data across participants. We found significant correlations between P3 and OC and SPL, while the correlation between MMN and early OC was only marginally significant after corrections for multiple comparisons. Nevertheless, these results might support the idea that MMN and P3 are associated with different hierarchical levels of deviance processing in the brain, with P3 related to both occipital and parietal activation. However, the correlations for the mechanisms were not significant, and no conclusive evidence for the null hypothesis was found either. Thus, the correlation data suggest that participants with a high prediction error effect in the P3 or a high adaptation effect in the vMMN do not necessarily have a high prediction error or adaptation effect in fMRI clusters. This could be a true phenomenon, suggesting that interindividual variability in adaptation- and PE-related processes in ERP and fMRI data is unrelated. However, this could also be due to varying neuronal sources across participants, despite some shared clusters. Furthermore, additional noise in the EEG data, which was acquired under challenging conditions in the scanner, may affect the variability of difference waves related to the underlying mechanisms of mismatch responses across participants. Future studies that allow for computational modeling e.g.,^31^ might be helpful in relating EEG and fMRI, depending on the mechanisms underlying deviance processing within participants.

In summary, we observed deviance-related effects in EEG as well as fMRI. The processes predominantly responsible for these effects varied systematically. In fMRI, we detected prediction-related activity in occipital and parietal areas that are part of the visual processing hierarchy and only a small adaptation-related effect in posterior visual cortices, which is in line with a hierarchical predictive processing account in visual deviance processing. Correspondingly, in the EEG, the vMMN was driven by adaptation, and the P3 was driven by a prediction error. The results highlight that the predominant mechanism underlying mismatch processing evolves during the course of information processing, with earlier time steps in sensory areas relying on adaptation and later time points in higher-order areas relying on a prediction error.

### Limitations of the study

While our study has many strengths, there are also limitations. We only investigated mechanisms of deviance processing during a condition where oddball stimuli had to be attended to but were not targets. Mechanisms predominantly observed during deviance processing might be modulated by task settings, as discussed above. Furthermore, we only used an equiprobable control condition to separate the mechanisms of deviance processing. Our results should be confirmed with other forms of control conditions e.g., cascade control^23^; or computational modeling approaches.^31^^,^^55^ The latter analytical focus would also allow a better coupling of fMRI and EEG data based on model estimates.

## Resource availability

### Lead contact

Further information and requests for resources and reagents should be directed to and will be fulfilled by the Lead Contact, Insa Schlossmacher (insa.schlossmacher@uni-muenster.de).

### Materials availability

This study did not generate new unique reagents.

### Data and code availability

• Data from this study have been deposited on the Open Science Framework (OSF) and is publicly available as of the date of publication. The DOI is listed in the key resources table.
• All original code has also been deposited on the OSF and is publicly available as of the date of publication. The DOI is listed in the key resources table.
• Any additional information required to reanalyze the data reported in this article is available from the lead contact upon request.

## Acknowledgments

We would like to thank all participants for their time and effort.

## Author contributions

I.S.: conceptualization, investigation, formal analysis, methodology, software, visualization, and writing – original draft. I.P.: formal analysis, investigation, and writing – review and editing. J.D.: formal analysis, investigation, and writing – review and editing. D.H.: investigation, software, and writing – review and editing. T.D.: methodology, software, and writing – review and editing. A.P.: methodology and writing – review and editing. M.-L.R.-P.: investigation and writing – review and editing. R.M.: investigation and writing – review and editing. M.B.: conceptualization, methodology, supervision, and writing – review and editing. T.S.: conceptualization, methodology, supervision, and writing – review and editing. All co-authors have read and approved the final version of the article.

## Declaration of interests

The authors declare no competing interests.

## STAR★Methods

### Key resources table

**Table:** 

REAGENT or RESOURCE	SOURCE	IDENTIFIER
**Deposited data**		

current study data	study cohort	OSF: https://doi.org/10.17605/OSF.IO/DX3GU

**Software and algorithms**		

MATLAB 2024b	MathWorks	RRID:SCR_001622
Statistical Parametric Mapping (SPM12)	Wellcome Department of Imaging Neuroscience, London, UK	RRID:SCR_007037
R Version 4.4.2	R Foundation	RRID:SCR_001905
Psychophysics Toolbox 3	http://psychtoolbox.org/	RRID:SCR_002881
MRIcroGL 2022-08-04	https://www.nitrc.org/projects/mricrogl	RRID:SCR_024413
Brain Vision Analyzer 2.2	BrainProducts	RRID:SCR_002356
FieldTrip	https://www.fieldtriptoolbox.org/	RRID:SCR_004849
DPABI	https://rfmri.org/DPABI	RRID:SCR_010501
Rfxplot	https://rfxplot.sourceforge.net/index.html	RRID:SCR_027375
original code	this paper	OSF: https://doi.org/10.17605/OSF.IO/DX3GU

### Experimental model and study participant details

Fifty-nine right-handed participants with normal or corrected-to-normal vision and no history of neurological or psychiatric illness took part in the experiment and were compensated with €10/h. Two participants had to be excluded due to excessive head movements (>3 mm) during recording, and one participant because of anomalies detected in the anatomical MRI. Furthermore, one participant had to be excluded due to EEG malfunctions, and one participant due to noncompliance with the experimental task. The remaining 54 participants (38 female, 16 male) were aged from 18 to 33 (M = 23.20, SD = 3.04). All participants provided informed consent and ethics committee of the Westphalia-Lippe Medical Association and the University of Münster approved the study (2019-049-f-S).

### Method details

#### Experimental procedure and stimulus material

Stimuli consisted of five different geometrical shapes, including a vertical rectangle, horizontal rectangle, square, circle, and diamond. The stimulus duration was 100 ms, and the shapes were gray (RGB [109, 109, 109]). All shapes were matched in surface area, which amounted to a radius of 1.5° for the circle, a side length of 1.33° for the square and diamond, as well as a side length of 1.88° and 0.94° for horizontal and vertical rectangles. A LCoS (Liquid Crystal on Silicon) projector (DLA-RSxx, JVCKenwood USA Corporation, USA) projected the image onto a semitransparent screen positioned at the front end of the scanner. Participants viewed the screen through a mirror attached to the head coil. In oddball blocks, the vertical and horizontal rectangles served as deviant and standard, counterbalanced across participants. The probability of deviant relative to standard stimuli was 20:80, and stimulus presentation was pseudorandomized so that no two deviants were presented consecutively. In control blocks, all five geometrical shapes were presented randomly with a probability of 20% (see Figure 1B). Throughout, the participants’ task was to respond via button press to target stimuli, which consisted of the above-described shapes with a longer duration (300 ms instead of 100 ms), which were randomly interspersed in the sequence. In total, 40 targets were included. Their frequency conformed to the stimulus probability of the current block, i.e., all geometrical shapes could be potential targets. In detail, in the control block, targets consisted of 20% vertical rectangles, 20% horizontal rectangles, 20% squares, 20% circles, and 20% diamonds. In oddball blocks, targets were 80% standards and 20% deviants. Interstimulus intervals for all stimuli, regardless of shape, ranged from 1.04 to 17.25 s (M = 3.01, SD = 2.06) and were derived using optseq2.^67^ Four different optseq sequences were computed and randomly assigned to two runs per participant, creating 12 different sequence combinations. In total, two runs of 250 stimuli (ca. 13 min each) were acquired and separated by a short break. One run could start with either an oddball or control block, which seamlessly changed to a control or oddball block in the middle of the run. Thus, each run consisted of 125 stimuli of an oddball sequence (∼6.5 min) and 125 stimuli of a control sequence (∼6.5 min). Whether the participants started with the oddball or control block was counterbalanced across participants. The second run was always presented in reverse order, e.g., if the first run was oddball-control, the second run was control-oddball. Before starting the experiment, a short practice block of about 1 min was presented in order to accustom the participants to the task. Stimuli in this practice block were structured like the experimental block that followed, i.e., they also included brief oddball and control sequences. At all times, a white fixation cross was presented on a black screen, and participants were asked to fixate during the run. Stimulus presentation and response collection were controlled by the software Presentation (Version 21.1, Neurobehavioral Systems, Albany, CA).

#### Data acquisition and preprocessing

##### FMRI

A 3-Tesla Siemens Magnetom Prisma with a 20-channel Siemens Head Matrix Coil (Siemens Medical Systems, Erlangen, Germany) was used to acquire MRI data. In a first step, we obtained a high-resolution T1-weighted scan with 192 slices for anatomical localization and coregistration (repetition time (TR) = 2130 ms, echo time (TE) = 2.28 ms, flip angle (FA) = 8°, field of view (FOV) = 256 × 256 mm, voxel size = 1 × 1 × 1 mm). A shimming field was applied in order to minimize magnetic field inhomogeneity. Then, we recorded two functional datasets per participant (2 runs) consisting of 353 volumes and 42 slices each by means of a T2∗-weighted echoplanar sequence sensitive to blood oxygenation level-dependent (BOLD) contrast (TR = 2300 ms, TE = 30 ms, FA = 90°, FOV = 216 × 216 mm, voxel size = 3 × 3 × 3 mm).

Preprocessing relied on SPM12 v7771 (Wellcome Department of Cognitive Neurology, London, UK) and the Data Processing & Analysis of Brain Imaging (DPABI) 4.3 toolbox^68^ in MATLAB (version R2019b; MathWorks Inc., Natick, MA; http://www.mathworks.com). We removed the first five data volumes to account for spin saturation effects. Then, slice-scan-time correction and realignment using a six-parameter (rigid body) linear transformation was performed. In the next step, we co-registered anatomical and functional images and segmented these into gray matter, white matter, and cerebrospinal fluid. Finally, we normalized functional data to Montreal Neurological Institute (MNI) standard space using DARTEL,^69^ resampled it to 3 mm isotropic voxels, and spatially smoothed it with an 8 mm full width at half maximum Gaussian kernel.

##### EEG

We recorded EEG data from a 32-channel MR-compatible EEG cap (BrainCap MR, Brain Products, Gilching, Germany), including an additional electrode at the subjects’ back, which monitored their electrocardiac activity. Impedances were kept below 10 kΩ using electrode gel (Abralyt HiCl, EasyCap GmbH, Herrsching, Germany), and EEG was recorded with a sampling rate of 5000 Hz and an online band-pass filter of 0.016–250 Hz. The BrainAmp MR amplifier (Brain Products) was positioned behind the participant’s head and connected to a computer in the console room via a fiber optic cable. A SyncBox (Brain Products) was used to synchronize the scanner gradient and the EEG acquisition system clocks. A photodiode in the scanner room connected to a BrainAmp ExG MR amplifier (Brain Products) was used to correct any delays between trigger and stimulus presentation later.

For preprocessing the raw EEG, first the scanner gradient artifacts and cardioballistic artifacts were corrected, and the sampling rate was reduced to 250 Hz in BrainVision Analyzer 2.1 (Brain Products). The data was then imported into Fieldtrip for further preprocessing. Data was band-stop filtered at 49–51 Hz (roll-off: −24 dB/octave) and at harmonic frequencies (up to 199–201 Hz) in order to minimize line noise. A 0.1 Hz high-pass filter (roll-off: −12 dB/octave) removed slow drifts. Then, the EEG signal was segmented into epochs of 200 ms before and 600 ms after stimulus onset. Using an independent component analysis (ICA), horizontal and vertical eye movement artifacts were removed from the data. Trials containing muscle artifacts and electrode jumps were manually excluded based on visual inspection, and bad channels were interpolated by using a weighted average of all neighbors. Data was re-referenced to a common average reference, and all trials were baseline-adjusted using the average of a pre-stimulus interval from −200 to 0 ms. Finally, we computed averages for the deviant, standard, and control stimulus. We computed the signal-to-noise ratio (SNR, computed by dividing the squared average over trials by the variance) for the standards for every sample in order to exclude participants with insufficient data quality (average SNR<0.02).

### Quantification and statistical analysis

#### fMRI

A general linear model (GLM) was estimated for each participant in the first-level analysis. In order to eliminate slow signal drifts, we used a high-pass filter with a cutoff of 128 s. We applied SPM’s pre-whitening method FAST^70^ to model autocorrelations as recommended by Olszowy and colleagues.^71^ The GLM design matrix contained the onsets of deviants, standards, controls, targets, and responses as predictors as well as six head movement parameters and a constant, which represented predictors of no interest. We included two predictors accounting for the stimuli presented during the control condition. One used the onsets of the stimulus physically identical to the deviant (later compared with the deviant and standard); the other modeled the onsets of all other control stimuli and was included as a nuisance regressor. These onsets were then convolved with a 2-gamma hemodynamic response function to model the BOLD signal change for each predictor. Contrast images (deviant – standard, deviant – control, control – standard) of the beta estimates were created for each participant for the second-level analysis.

In order to isolate mismatch-related activity in the second-level analysis, we used cluster-based permutation tests as implemented in PALM.^72^ The voxel-wise α amounted to 0.005; a cluster was deemed significant with α < 0.05. The number of permutations was set to 10000. Based on the meta-analysis of Kim,^1^ the following areas of interest were identified and included in one mask based on the Harvard Oxford Atlas^73^: Occipital cortex (OC), anterior cingulate cortex (ACC), supplementary motor area (SMA), inferior frontal junction (IFJ), inferior and superior parietal lobule (IPL/SPL), temporo-parietal junction (TPJ), insula and thalamus (Figure 1C). Areas were chosen to correspond to the modality and task of the current study visual and task-irrelevant, see Kim^1^ for details. In the second step, we built upon the results of the first analysis (deviant – standard) and used significant clusters as a mask for the cluster-based permutation tests of adaptation-related activity (control – standard) and prediction error-related activity (deviant – control). In order to analyze potential null effects in fMRI, we extracted average beta values from 8 mm spheres around peak coordinates reported in the meta-analysis of Kim^1^ and computed Bayes Factors (BF), with BF_01_ denoting the evidence for the null hypothesis and BF_10_ the evidence for the alternative hypothesis. We use the conventions from Keysers,^74^ which are based on Jeffreys,^75^ to interpret the results of our Bayesian analyses.

In order to visualize event-related time courses, we extracted activity around 8 mm spheres at peak voxels of the significant clusters using the rfxplot toolbox in SPM.^76^ Activation maps were visualized using MRIcroGL.^77^

#### EEG

We used an averaged amplitude approach to investigate early and late mismatch responses. For the vMMN, we averaged the amplitude around the N1 peak of deviant, standard, and control at electrodes P7 and P8 between 160 and 210 ms. For the P3, we averaged the amplitude of deviant, standard, and control at centro-parietal electrodes (C3, Cz, C4, CP5, CP1, CP2, CP6, P3, Pz, P4) between 300 and 600 ms. We conducted one-sided *t*-tests to investigate whether mismatch responses, adaptation-related or prediction-error-related activity could be observed. We additionally report Bayes Factors (BF).

#### Integration of EEG-fMRI

We calculated Pearson correlation coefficients (r) to test whether we could link the inter-subject variability of the EEG and fMRI responses, both for the main effects of mismatch responses and the identified mechanisms. Specifically, we correlated differences in EEG amplitudes (e.g., deviant – standard) averaged across intervals and electrodes of interest (e.g., vMMN) with differences in beta estimates (e.g., deviant – standard) averaged across voxels of significant clusters. We used false discovery rate (FDR) correction^78^ to account for the multiple comparison problem posed by the 12 tested correlations. Again, we additionally report Bayes Factors.
